# Some Thoughts on Oral Surgery of Interest to the Physician

**Published:** 1912-05

**Authors:** Robin Adair

**Affiliations:** Atlanta, Ga.; Professor Alveolar Pyorrhea at Southern Dental College


### SOME THOUGHTS ON ORAL SURGERY OF INTEREST TO THE PHYSICIAN.

By Robin Adair, M. D., D.D. S., Atlanta, Ga.

*Professor Alveolar Pyorrhea at Southern Dental College.*

A Philadelphia doctor has figured out that a man's jaw travels 107 miles in the task of eating and talking in an average life of 70 years. The lengthy journey of a woman's jaw has not yet been computed. However, a man with a scolding wife says he has less fear of the jaws of death than the jaws of life. As the jaws and their surroundings compose Oral Surgery, I approach this subject with much deliberation and fear.

A few years ago, our Dental colleges gave little or no instructions in this branch with the idea that work of this character belonged entirely to the M. D. To-day every well equipped dental college claims this chair of prime importance, and it is being hoped that in the near future oral surgeons will be so equipped that the specialist and general practitioner will become better acquainted with their work, and express confidence by consultation or referring all cases about the mouth to them for operation. The general practitioner should feel the same about this specialty as he does the operations on the nose by the rhinologist, or the operations on the eye by the ophthalmologist. From the fact that surgery is so intimately connected with the mechanical procedures, the training of the dentist fits him to be of great assistance when it comes to making appliances for surgical work. The further fact that the dentist is an adept at minute diagnosis, coupled with his mechanical and pathological training, fits him to undertake if he choose, the specialty of oral surgery.

The specialty of oral surgery is at present without any well-defined lines as to its limit of practice, and not so well understood by either the professional ranks or the laity. The writer in his capacity of oral surgeon to Grady Hospital, has found himself either trying to do too much or not enough, either direction leading to criticism.

A dentist cannot practice oral surgery without the co-operation of the M. D., and it is equally true that the oral surgeon can be of the greatest aid to the M. D. in their work, which in more cases than they have probably thought, or either caused, or associated with lesions about the mouth.

### SEPARATION OF THE MAXILLA.

As an illustration of this thought, the well-established custom as a remedy for stenosis of deflected nasal septum is the removal of turbinates. The oral surgeon has suggested expanding the maxilla, to remove the pressure against the septum, and at the same time to enlarge the base of the nose. In favorable cases the septum becomes straight, the turbinate bones will return to the normal, and circulation has improved as well as the regular nasal inspirations. Mental activity as well as smell are also restored. In those cases where deafness is caused by pressure on the eustachian tubes, this method has brought permanent relief in a short time. The appliance made by the oral surgeon is simple and worn with comparative comfort by the patient.

Some affections of the eye caused by the clogging of the tear ducts, have been relieved by this procedure, and reduces, as it does inflammation of the condition of the mucous membrane, allowing free drainage.

This operation is now beginning to receive its just dues, and at the next meeting of the eye and nose specialists in Philadelphia, a symposium has been given over to this subject. The most enthusiastic advocate of the separation of the maxilla is Dr. G. V. I. Brown, of Milwaukee, Wis. At the present time, Dr. Brown is proving his case by interesting experiments on dogs, in that he produces a simple nasal deflected septum and congested nasal membrane. It is interesting also to note in this connection that whereas the nasal passages of the dog are nearly germ proof, in this changed state they are easily susceptible to infection.

### TRIGEMINAL NEURALGIA.

Local influences in the mouth are of greatest importance in the proper diagnosis for the treatment of neuralgias about the face, and yet this very importance has lead to dentists claiming more credit than was probably due, inasmuch as sometimes the extraction of a tooth has corrected these conditions. While dentists are prone to overlook the vital features and pathological con-oral surgeon can have better success with these procedures, in ditions accompanying these pains, only those who are familiar with conditions of the teeth can be expected to diagnose or treat cases which do come from dental origin. These are the cases which should be worked out by consultation between the physician and the oral surgeon.

The writer once had a patient, who was a great sufferer for many years, and a radical operation was advised by her physician as her only relief. In making a careful examination of the mouth, I removed a crown, suspecting trouble beneath. My expectation was realized in that I removed a gold wire one-half inch long, which had protruded through the apex of root of the tooth to the floor of the nose. Relief was immediate. On the other hand, I have had several cases where all my endeavors could not place the responsibility to dental origin, again showing that close relationship and benefit can be derived by associated diagnosis.

### Fractures of the Jaw.

Operations seem to run in cycles. Of late the writer has treated quite a number of fractures of the jaw. As the treatment differs somewhat from that of the general surgeon, I herewith give some of the methods used in treatment.

In these operations the oral surgeon can be of great aid to the general surgeon. The latter seldom pays any attention to the occlusion of the teeth just so the jaw is united being sufficient. On the other hand, the oral surgeon holds that articulation is of prime importance and the jaw united with the teeth thrown out of commission, is depriving the patient of all his rights to food mastication. It is not the intention of the writer to go into any classification or detail description of these cases, except insofar as they are applicable to certain classes of fractures which the oral surgeon treats in a different way from the general practitioner.

In compound and multiple fractures, wiring of the fragments of the bones now seems to be the most popular method. The general surgeon in performing these operations uses too large a drill and frequently drills through the roots of the teeth. The that he knows the exact position of the teeth in the jaws, and can manipulate a smaller drill and wire. (Illustrated by models.)

Another method which has been used from time immemorial, is to approximate the fractured ends by wiring the teeth of the injured jaw, and even wiring the fractured jaw to the sound opposing one. The dentist has recently reviewed this procedure, and developed a manipulation of wire which is most reliable in simple fractures of the jaw. (Illustrated by model.)

The advantage of this method is that the jaw can be released in a few moments and again be brought in occlusion, and also that the teeth can be kept in a clean condition. The prospect of keeping the jaws united for a set occlusion, has raised some objection, but in practice this has not been so. The patient is most comfortable and can go among his friends without any embarrassment. Experience has also taught us that the question of feeding is one of considerable ease to the patient. The angle fracture band, always to be had on short notice, is another method we frequently use. These require the operator to be familiar with the method. The bands are cemented on sound teeth on either side of the break in the injured jaw, when the retention screw is turned, bringing the fractured ends together. If the case require, more bands are used on opposing jaw, binding the jaws together in proper occlusion.

Several models will be shown of cases of gun shot origin, where is was impossible to bring the fractured parts of the bones together without causing a disturbance of the occlusion and shortening of the jaws. It became necessary to place the parts in normal position by means of the interdental splint, the appliance shown being of original design. In this case, we trusted nature to form new bone, or the forming of fibrous tissue, and with the aid of permanent "bridge splints" will give a serviceable jaw.

The oral surgeon is prepared and speedily guards against oral sepsis, which is of the greatest importance when we bear in mind that the mouth is a hotbed of so many varieties of germs. To his special regard of oral hygiene in these cases, is due much of the marvelous success accomplished.

This paper only deals with a few points to secure interest between the oral surgeon and the general practitioner of medicine, enough I hope, to show that a closer connection will be to our mutual advantage.

